# A fatal case of Rosai–Dorfman disease

**DOI:** 10.1002/ccr3.1036

**Published:** 2017-06-15

**Authors:** Daniel Bulyashki, Zarina Brady, Shahswar Arif, Nikolay Tsvetkov, Radoslav Stoyanov Radev

**Affiliations:** ^1^ Department of Thoracic Surgery Saint Marina Hospital Varna Bulgaria; ^2^ Meditsinski Universitet Varna Prof Dr Paraskev Stoyanov Varna Bulgaria; ^3^ Faculty of Medicine Medical University of Varna Varna Bulgaria

**Keywords:** Emperipolesis, fatal, Lymphodenopathy, nonspecific symptoms, Rosai–Dorfman disease

## Abstract

As Rosai–Dorfman Disease presents generally nonspecific symptoms, differential diagnosis can be of great learning curve for physicians. Additionally, RDD does not usually threaten life and spontaneous remission is frequently observed. However, unusually in our case the patient passed away within 1 month of confirmed diagnosis.

A 38‐year‐old male was admitted with a fever (39°C), abdominal and back discomfort, and extensive lymphadenopathy. Laboratory tests revealed progressive thrombocytopenia and leukocytosis, platelet count of 84 × 109/L and peak WBC of 46.52 × 109/L. CT scanning uncovered enlarged lymph nodes of the neck, axillary (28/23 mm), hilar (21/24 mm), subcarinal (19/18 mm), paraaortic (30/29 mm), mesenteric (41/35 mm) and curvatura minor gastritis (26/22 mm). Additionally, abdominal ultrasound revealed free fluid in the abdomen combined with an enlarged liver (157 mm) and spleen (157/56 mm). Sterile blood culture ruled out the possibility of a bacterial or fungal infection. An excisional biopsy of an axillary lymph node was performed with no complications. Microscopic examination of the specimen revealed a diffuse proliferation of histiocytes, fibrosis with inflammatory infiltration of the node's capsule and lymphocytes engulfed in the cytoplasm of the histocyte‐like cells (emperipolesis), typical pathology of Rosai–Dorfman 1.

Immunohistochemical stain analysis revealed histiocytes staining strongly for S‐100 protein, CD68 protein, and expansion of sinusoids with an uncontrolled expression of plasma cells, lymphocytes and histiocytes with large oval nuclei. Despite the disease having relatively good prognosis, the patient was dead within a month of confirmed diagnosis, occurring in <3% of Rosai–Dorfman disease cases. The patient's condition progressively deteriorated prior to death. Enlargement of lymph nodes, acute circulatory deficiency, and acute respiratory failure was treated with Dexamethasone and Methylprednisolone. The cause of death was due to suspected respiratory infections resulting from therapy‐based immunosuppressants and initial chemotherapy treatment. No autopsy was carried out due to the patients family declining. As observed in our patient, common symptoms of Rosai–Dorfman disease are nonspecific and can be manifested in malignant and nonmalignant diseases, such as lymphomas, Langerhans cell histiocytosis 2, tuberculosis and sarcoidosis, creating a potential for misdiagnosis (Figs 1, 2, 3).

**Figure 1:**
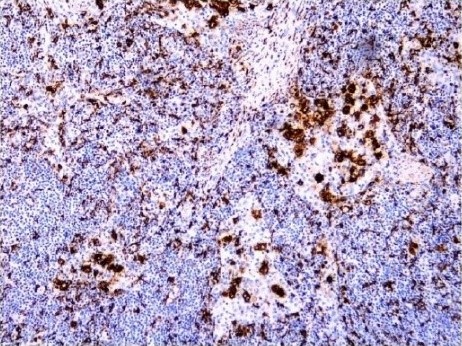
Immunohistochemical analysis showing histiocytes staining strongly for CD68 protein, indicating the presence of lymphocytes within cytoplasmic vacuoles. (Magnification X40; CD68).

**Figure 2:**
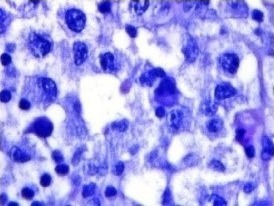
Expansion of sinusoids with an uncontrolled expression of plasma cells, lymphocytes, and histiocytes with large oval nuclei. (Magnification X400; Haematoxylin stain).

**Figure 3:**
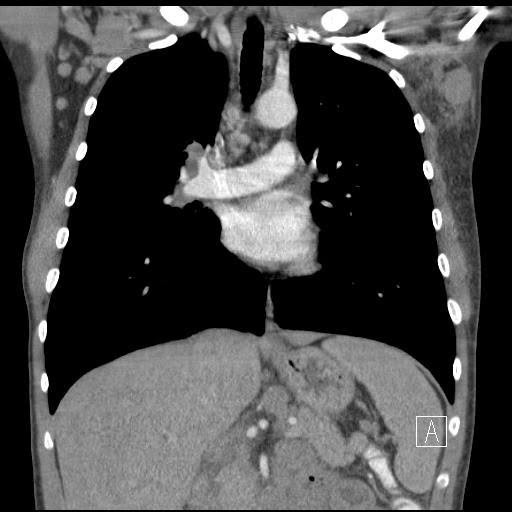
CT scan showing axillary lymph nodes.

## Conflict of Interest

None declared.

## Authorship

DB: primary surgeon of the excisional biopsy; involved in critical analysis of report. ZB: wrote the report. SA: wrote the report. NT: surgeon of the excisional biopsy of lymph nodules. RR: involved in the final approval of report.
